# Controlled state transfer in a Heisenberg spin chain by periodic drives

**DOI:** 10.1038/s41598-018-31552-w

**Published:** 2018-09-10

**Authors:** H. J. Shan, C. M. Dai, H. Z. Shen, X. X. Yi

**Affiliations:** 1 Center for Quantum Sciences, Northeast Normal University, Changchun, 130117 China; 2 Center for Advanced Optoelectronic Functional Materials Research, and Key Laboratory for UV Light-Emitting Materials and Technology of Ministry of Education, Northeast Normal University, Changchun, 130024 China

## Abstract

The spin chain is a system that has been widely studied for its quantum phase transition. It also holds potential for practical application in quantum information, including quantum communication and quantum computation. In this paper, we propose a scheme for conditional state transfer in a Heisenberg *XXZ* spin chain. In our scheme, the absence or presence of a periodic driving potential results in either a perfect state transfer between the input and output ports, or a complete blockade at the input port. This scheme is formalized by deriving an analytical expression of the effective Hamiltonian for the spin chain subject to a periodic driving field in the high-frequency limit. The influence of the derivation of the optimal parameter on the performance of the state transfer is also examined, showing the robustness of the spin chain for state transfer. In addition, the collective decoherence effect on the fidelity of state transfer is discussed. The proposed scheme paves the way for the realization of integrated quantum logic elements, and may find application in quantum information processing.

## Introduction

One important task of quantum information processing is to transfer quantum states from one location (A) to another (B). In quantum communication scenarios, this is quite clear, since the goal is communicate between A and B^1–4^ with quantum states as the information carrier. In quantum computation, good communication between different parts of quantum computer is also essential^5,6^. There are various physical systems that can be used as quantum channels to transfer quantum states, one of which is the quantum spin system. This system can be defined as a collection of interacting quantum bits on the graph, whose dynamics are governed by an appropriate Hamiltonian.

On the other side, spin field effect transistors originally proposed in 1990 by Supriyo Datta and Biswajit Das^7,8^ is an improved design on the traditional transistor invented in the 1940 s. The spin transistor comes about as a result of research on the ability of electrons to naturally exhibit one of two (and only two) states of spin: known as spin up and spin down. Unlike its classical counterpart, which operates on an electric current, spin transistors operate on electrons on a more fundamental level. It is essentially the application of electrons set in particular states of spin to store information. Since the spin field-effect transistors (or spin transistors) have low power consumption and high speed operation that can be applied to logic circuits. In addition, quantum spin transistor can be applied to control state transfer between two sites. The spin chain of Heisenberg types^9–11^ has been realized in quantum dots^12,13^, molecular magnets, Josephson junction arrays. The physics of driving quantum system has been studied extensively for decades^14–28^, in particular the research of such systems is of great significance to quantum information processing^29–34^ in recent years. With these knowledge, we wonder if a controlled state transfer can be realized in a spin chain by a periodic drive. Except the perfect state transfer, the completely state blockage is also interesting, since it is similar to quantum memory^35,36^ when the transmission is blocked. Of course, periodic driving is only a way to control the transfer of quantum states. Other methods to control the state transfer in this direction include acceleration of adiabatic transfer by using dressed states^37^, control quantum state transmission in an array of optomechanical cells^38^, robust control^39,40^ and so on.

Quantum communication over short distances through a spin chain, in which adjacent qubits are coupled with equal strength has been studied in detail^41^. The propagation of quantum information in rings has also been studied^42,43^. And near perfect state transition was obtained for uniform couplings provided that a spatially varying magnetic field was introduced^44^. At the same time, the perfect state transfer has been studied, without periodic driving^45,46^. The previous schemes have made so many approximations to realize the perfect transmission, which inspire us to find a more precise method, using a dynamic Heisenberg spin chain, to achieve the perfect state transfer with high fidelity and short time as well as fewer approximations.

In our work, we propose a scheme to realize the state transfer from the input port (target spin) to the output port. This transfer can be controlled by a periodic drive reminiscent of the transistor in electronics. We find that the state transfer can be performed if there are no period driving field in the gate, while the transfer is completely blocked if the gate(the spin in the middle) experiences a period driving. We achieve quantum state transmission and blockade while minimizing dynamics control, and the accuracy of both state transmission and blockade can be increased by the control. The analytical solution of the particle transport condition and the sensitivity of spin chain are also derived and analyzed, and which are applied to study the effect of the fluctuation of system parameters on state transmission and blockade. Furthermore, we also investigated the effect of decoherence on the state transfer.

The remainder of this paper is organized as follows. First, we introduce our model that consists of a periodically driven spin $-\frac{1}{2}$ particle coupled a one-dimensional Heisenberg spin chain. We discuss the case of *N* = 4 and derive an analytical expression for the perfect state transfer. Then, we analyzed the condition of blocking the particle state transmission by an added driving. An stochastic Hamiltonian is used to simulate the influence of classical noise on the quantum system, which shows the effect of decoherence on the state transfer. Finally, we made a brief analysis of *N* particles and present some discussions and conclusions.

## Methods

### System and Effective Hamiltonian

**Figure 1:**
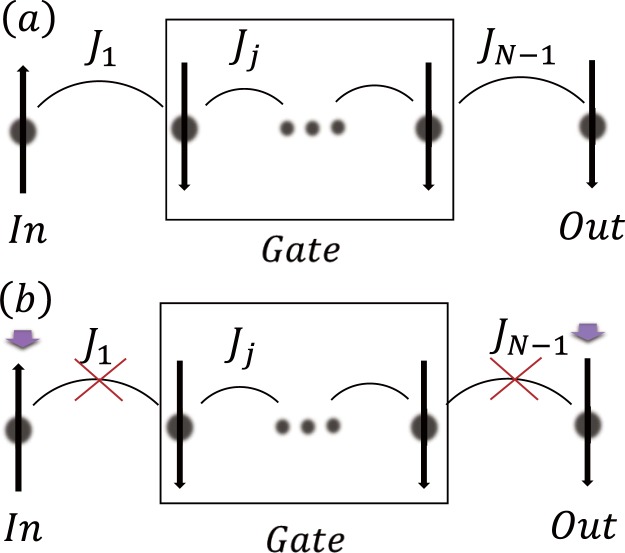
(**a**) The ‘In’ and ‘Out’ ports are coupled with the central gate region with coupling constants coefficient *J*_1_ and *J*_*N*−1_, respectively. Resonant transfer of a spin excitation between the input and output ports occurs without periodic driving. (**b**) The spin excitation transfer is blocked if a special periodic driving is applied.

We present a scheme for a quantum spin transistor realized with a Heisenberg spin chain. Our quantum spin transistor operates at an arbitrary spin state *α*|↓〉 + *β*|↑〉 at the input port, if there are no periodic driving field. And the presence of a periodic driving field can block the transfer of the spin state. As shown in Fig. 1 the input and output ports for the spin state are represented by the *j* = 1 and *j* = *N* sites of the chain and the input and output ports are coupled with coupling strength *J*_1,*N*−1_. Consider a one-dimensional *XXZ* spin chain subject to a periodic driving *H*_1_(*t*). The Hamiltonian of the dynamics system is *H*(*t*) = *H*_0_ + *H*_1_(*t*), with

1 $${H}_{0}=\sum _{j=1}^{N}{h}_{j}{\sigma }_{z}^{j}-\frac{1}{2}\sum _{j=1}^{N-1}{J}_{j}[{\sigma }_{x}^{j}{\sigma }_{x}^{j+1}+{\sigma }_{y}^{j}{\sigma }_{y}^{j+1}+{\rm{\Delta }}{\sigma }_{z}^{j}{\sigma }_{z}^{j+1}],$$

2 $${H}_{1}(t)=\sum _{j=1}^{N}\,{f}_{j}(t){\sigma }_{z}^{j},$$

where ${\sigma }_{x,y,z}^{j}$ are the Pauli matrices with *j* = 0, 1, ..., *L*, respectively, labeling the system spin and the spins in the chain, *N* is the total number of sites in the spin chain; *h*_*j*_ is the local magnetic field, *f*_*j*_(*t*) is a periodic driving only on the system, *J*_*j*_ are the coupling strength between the nearest neighbors and Δ is the asymmetry parameter. To proceed further, it is clear that we can focus on the sing-particle subspace (|1〉 = |100⋅⋅⋅0〉, |2〉 = |010⋅⋅⋅0〉,…), where |*m*〉, *m* = 1, 2, 3, ..., *N* refers to a state with a single excitation at site *m*. We shall adopt the convention that |0_*j*_〉 (|1_*j*_〉) represents the spin-down (spin-up). Our goal is to transfer population from the initial state (|1〉 = |10⋅⋅⋅00〉) to the final state (|*N*〉 = |00⋅⋅⋅01〉) and the complete blocking of the state transmission. To make the problem more convenient, we make a frame rotating via $\tilde{H}(t)={U}_{1}^{\dagger }H(t){U}_{1}-i{U}_{1}^{\dagger }{\partial }_{t}{U}_{1}$, and ${U}_{1}={\mathscr{T}}\,\exp [-i{\int }_{0}^{t}{H}_{1}(\tau )d\tau ]$, where ${\mathscr{T}}$ is the time-ordering operator. The Hamiltonian at any time *τ*, *H*_1_(*τ*), commutes with each other. In other words, we can obtain the identity [*H*_1_(*t*_1_),*H*_1_(*t*_2_)] = 0 for $\forall ({t}_{1},{t}_{2})$. Therefore, *H*(*t*) can be rewritten as $\tilde{H}(t)={U}_{1}^{\dagger }{H}_{0}{U}_{1}$. Then the Hamiltonian becomes

3 $$\begin{array}{rcl}\tilde{H}(t) & = & \sum _{j=1}^{N}{h}_{j}{\sigma }_{z}^{j}-\frac{1}{2}\sum _{j=1}^{N-1}{J}_{j}[\cos (2{F}_{j+1}(t)-2{F}_{j}(t))({\sigma }_{x}^{j}{\sigma }_{x}^{j+1}+{\sigma }_{y}^{j}{\sigma }_{y}^{j+1})\\  &  & +\,\sin (2{F}_{j+1}(t)-2{F}_{j}(t))({\sigma }_{y}^{j}{\sigma }_{x}^{j+1}-{\sigma }_{x}^{j}{\sigma }_{y}^{j+1})+{\rm{\Delta }}{\sigma }_{z}^{j}{\sigma }_{z}^{j+1}],\end{array}$$

where ${F}_{j}(t)={\int }_{0}^{t}{f}_{j}(\tau )d\tau$. In the high-frequency limit, we arrive at the effective Hamiltonian by $\tilde{U}(T)={\mathscr{T}}$ $\exp [-i{\int }_{0}^{T}\tilde{H}(\tau )d(\tau )]=\exp [\,-i{H}_{eff}T]$^26^

4 $$\begin{array}{rcl}{H}_{eff} & = & \sum _{j=1}^{N}{h}_{j}{\sigma }_{z}^{j}-\frac{1}{2}\sum _{j=1}^{N-1}[{J}_{j}^{eff}({\sigma }_{x}^{j}{\sigma }_{x}^{j+1}+{\sigma }_{y}^{j}{\sigma }_{y}^{j+1})\\  &  & +{J}_{j}^{eff^{\prime} }({\sigma }_{y}^{j}{\sigma }_{x}^{j+1}-{\sigma }_{x}^{j}{\sigma }_{y}^{j+1})+{J}_{j}{\rm{\Delta }}{\sigma }_{z}^{j}{\sigma }_{z}^{j+1}],\end{array}$$

where ${J}_{j}^{eff}=\mathrm{1/}T{\int }_{0}^{T}{J}_{j}\,\cos (2{F}_{j+1}({t}_{1})-2{F}_{j}({t}_{1}))d{t}_{1}$, ${J}_{j}^{eff^{\prime} }=\mathrm{1/}T{\int }_{0}^{T}{J}_{j}\,\sin (2{F}_{j+1}({t}_{1})-2{F}_{j}({t}_{1}))d{t}_{1}$. To realize the desired spin transfer, we restrict that the spin chain to satisfy spatially symmetric ${J}_{j}^{eff}={J}_{N-j}^{eff}$, ${J}_{j}^{eff^{\prime} }={J}_{N-j}^{eff^{\prime} }$ and *h*_*j*_ = *h*_*N*+1−*j*_ with *h*_1_ = *h*_*N*_ = 0.

In this work, we mainly illustrate how the spin excitation initially localized at the left boundary site can be controlled to transfer to another side by periodic drives. We first consider the quantum dynamics of individual particles that can be transmitted from the left end of the boundary to the right side. The evolution operator reduces to $\tilde{U}(t)\approx {e}^{-i{H}_{eff}t}$, and with the initial state |1〉 we obtain the condition for perfect state transfer at *t*_*out*_, $\tilde{U}({t}_{out}\mathrm{)|1}\rangle =|N\rangle$. At the same time, we find a mirror operator $S={\sum }_{j=1}^{N}|j\rangle \langle N-j+1|$, and *S*|1〉 = |*N*〉. This means that a quantum state at site *j* can be transferred to its mirror-conjugate site *N* − *j* + 1. Since the Hamiltonian in Eq. (4) is mirror symmetrical, i.e., *S* commutes with *H*_*eff*_. Therefore, the condition for perfect state transfer is in the following form

5 $$(\sum _{j}^{N}({e}^{-i{H}_{eff}{t}_{out}}-S)|{{\rm{\Psi }}}_{j}\rangle \langle {{\rm{\Psi }}}_{j}|)\mathrm{|1}\rangle =0,$$

where |Ψ_*j*_〉 are the eigenstates of *H*_*eff*_ and *S*.

6 $$(\sum _{i}^{N}({e}^{-i{\lambda }_{j}^{eff}{t}_{out}}-{\varepsilon }_{j})|{{\rm{\Psi }}}_{j}\rangle \langle {{\rm{\Psi }}}_{j}|)\mathrm{|1}\rangle =0,$$

where the *ε*_*j*_ and ${\lambda }_{j}^{eff}$ are the eigenvalues of *S* and *H*_*eff*_. Since the values of *ε*_*j*_ are ±1,we change the above equation to $({\sum }_{i}^{N}({e}^{-i{\lambda }_{j}^{eff}{t}_{out}}-{(-1)}^{j+1})|{{\rm{\Psi }}}_{j}\rangle \langle {{\rm{\Psi }}}_{j}|)\mathrm{|1}\rangle =0$, and the condition for perfect state transmission is^10,11^

7 $$({\lambda }_{j+1}^{eff}-{\lambda }_{j}^{eff}){t}_{out}=\pi (2{m}_{j}+1),$$

where without loss of generality, we rearrange ${\lambda }_{j}^{eff}$ such that ${\lambda }_{j}^{eff} > {\lambda }_{i}^{eff}$ when *j* > *i*, which leads to *m*_*j*_ being a positive integer. We interpret it to be the signature of perfect communication (or perfect state transfer) between initial state and final state at time *t*_*out*_. As proven in ref.^47^, for a symmetric tridiagonal matrix, the symmetric substitution of eigenvectors because it is determined by the number of symbols changed (an odd number indicates that the eigenvector is antisymmetric). Our matrix *H*_*eff*_ is a three-diagonal matrix with positive coefficients ${J}_{j}^{eff}$ and ${J}_{j}^{eff^{\prime} }$. Considering that ${\lambda }_{j}^{eff}$ is a set of ordered eigenvalues, i.e., ${\lambda }_{j}^{eff} < {\lambda }_{j+1}^{eff}$, and the necessary and sufficient condition for state transition in symmetric chain is Eq. (7), the pairwise ratio of eigenvalues must be rational.

### The Scheme of *N* = 4 Spins with Three Kinds of Drives

We will illustrate the scheme using a chain of *N* = 4 spins with a single excitation. Now we give a detailed calculation for the perfect state transmission through the Heisenberg spin chain. First, we consider the absence of periodic driving field.

For *N* = 4 spin chain, the system has two different interaction coefficients *J*_1_( = *J*_3_) and *J*_2_, and we assume *h*_2_ = *h*_3_ = *h*. We set the system initially in the state |↑↓↓↓〉, which we aim to efficiently transfer to final state |↓↓↓↑〉. In the basis of {|↑↓↓↓〉, |↓↑↓↓〉, |↓↓↑↓〉, |↓↓↓↑〉}, the Hamiltonian matrix becomes

8 $${H}_{eff}=(\begin{array}{cccc}-2h-\frac{1}{2}{J}_{2}{\rm{\Delta }} & -({J}_{1}^{eff}+i{J}_{1}^{eff^{\prime} }) & 0 & 0\\ -({J}_{1}^{eff}+i{J}_{1}^{eff^{\prime} }) & \frac{1}{2}{J}_{2}{\rm{\Delta }} & -({J}_{2}^{eff}+i{J}_{2}^{eff^{\prime} }) & 0\\ 0 & -({J}_{2}^{eff}+i{J}_{2}^{eff^{\prime} }) & \frac{1}{2}{J}_{2}{\rm{\Delta }} & -({J}_{1}^{eff}+i{J}_{1}^{eff^{\prime} })\\ 0 & 0 & -({J}_{1}^{eff}+i{J}_{1}^{eff^{\prime} }) & -2h-\frac{1}{2}{J}_{2}{\rm{\Delta }}\end{array}).$$

The Hamiltonian reduces to

9 $${H}_{eff^{\prime} }=(\begin{array}{cccc}-2h-\frac{1}{2}{J}_{2}{\rm{\Delta }} & -{J}_{1} & 0 & 0\\ -{J}_{1} & \frac{1}{2}{J}_{2}{\rm{\Delta }} & -{J}_{2} & 0\\ 0 & -{J}_{2} & \frac{1}{2}{J}_{2}{\rm{\Delta }} & -{J}_{1}\\ 0 & 0 & -{J}_{1} & -2h-\frac{1}{2}{J}_{2}{\rm{\Delta }}\end{array}),$$

when the periodic driving is absent. Since *S* commutes with ${H}_{eff^{\prime} }$, the Hamiltonian matrix is block diagonal in the basis spanned by $\{\frac{1}{\sqrt{2}}(|\,\uparrow \,\downarrow \,\downarrow \,\downarrow \,\rangle +|\,\downarrow \,\downarrow \,\downarrow \,\uparrow \,\rangle )$, $\frac{1}{\sqrt{2}}(|\,\downarrow \,\uparrow \,\downarrow \,\downarrow \,\rangle +|\,\downarrow \,\downarrow \,\uparrow \,\downarrow \,\rangle )$, $\frac{1}{\sqrt{2}}(|\,\uparrow \,\downarrow \,\downarrow \,\downarrow \,\rangle \,-\,|\,\downarrow \,\downarrow \,\downarrow \,\uparrow \,\rangle )$, $\frac{1}{\sqrt{2}}(|\,\downarrow \,\uparrow \,\downarrow \,\downarrow \,\rangle \,-\,|\,\downarrow \,\downarrow \,\uparrow \,\downarrow \,\rangle )\}$,

10 $${H}_{eff^{\prime} }=(\begin{array}{cccc}-2h-\frac{1}{2}{J}_{2}{\rm{\Delta }} & -{J}_{1} & 0 & 0\\ -{J}_{1} & \frac{{J}_{2}({\rm{\Delta }}-\mathrm{2)}}{2} & 0 & 0\\ 0 & 0 & -2h-\frac{1}{2}{J}_{2}{\rm{\Delta }} & -{J}_{1}\\ 0 & 0 & -{J}_{1} & \frac{{J}_{2}\mathrm{(2}+{\rm{\Delta }})}{2}\end{array}).$$

We diagonalize the Hamiltonian and find that the eigenvalues of the inner block are

11a $${\lambda }_{1}^{eff^{\prime} }=\frac{1}{2}(-2h-{J}_{2}-\sqrt{4{J}_{1}^{2}+{(2h+{J}_{2}(-1+{\rm{\Delta }}))}^{2}}),$$

11b $${\lambda }_{2}^{eff^{\prime} }=\frac{1}{2}(-2h+{J}_{2}-\sqrt{4{J}_{1}^{2}+{\mathrm{(2}h+{J}_{2}\mathrm{(1}+{\rm{\Delta }}))}^{2}}),$$

11c $${\lambda }_{3}^{eff^{\prime} }=\frac{1}{2}(-2h-{J}_{2}+\sqrt{4{J}_{1}^{2}+{\mathrm{(2}h+{J}_{2}(-1+{\rm{\Delta }}))}^{2}}),$$

11d $${\lambda }_{4}^{eff^{\prime} }=\frac{1}{2}(-2h+{J}_{2}+\sqrt{4{J}_{1}^{2}+{\mathrm{(2}h+{J}_{2}\mathrm{(1}+{\rm{\Delta }}))}^{2}}).$$

Then, we bring the above eigenvalues into Eq. (7) and get the following solutions

12 $$\begin{array}{rcl}h & = & {h}_{0}\equiv \mathrm{1/2}{J}_{2}(({m}_{3}-{m}_{1})(2+{m}_{1}+2{m}_{2}+{m}_{3})/{(1+{m}_{1}+{m}_{3})}^{2}-{\rm{\Delta }}),\\ {J}_{1} & = & \mathrm{1/2}{J}_{2}\sqrt{((1+2{m}_{1})(1+2{m}_{2})(1+2{m}_{3})(3+2{m}_{1}+2{m}_{2}+2{m}_{3}))/{(1+{m}_{1}+{m}_{3})}^{4}}.\end{array}$$

Therefore, the eigenvalues of *H*_*eff*′_ can be rewritten into the following form

13 $$\begin{array}{rcl}{\lambda }_{1}^{eff^{\prime} } & = & \frac{1}{2}{J}_{2}(\,-\,(1+2{m}_{2})(1+2{m}_{3})/({(1+{m}_{1}+{m}_{3})}^{2}-\,2+{\rm{\Delta }}),\\ {\lambda }_{2}^{eff^{\prime} } & = & \frac{1}{2}{J}_{2}(\,-\,(1+2{m}_{2})(1+2{m}_{3})/({(1+{m}_{1}+{m}_{3})}^{2}-\,2+{\rm{\Delta }})+\frac{{J}_{2}(1+2{m}_{1})}{1+{m}_{1}+{m}_{3}},\\ {\lambda }_{3}^{eff^{\prime} } & = & \frac{1}{2}{J}_{2}(\,-\,(1+2{m}_{2})(1+2{m}_{3})/({(1+{m}_{1}+{m}_{3})}^{2}-\,2+{\rm{\Delta }})+\frac{2{J}_{2}(1+{m}_{1}+{m}_{2})}{1+{m}_{1}+{m}_{3}},\\ {\lambda }_{4}^{eff^{\prime} } & = & \frac{1}{2}{J}_{2}(\,-\,(1+2{m}_{2})(1+2{m}_{3})/({(1+{m}_{1}+{m}_{3})}^{2}-\,2+{\rm{\Delta }})+\frac{2{J}_{2}(\frac{3}{2}+{m}_{1}+{m}_{2}+{m}_{3})}{1+{m}_{1}+{m}_{3}}.\end{array}$$

**Figure 2:**
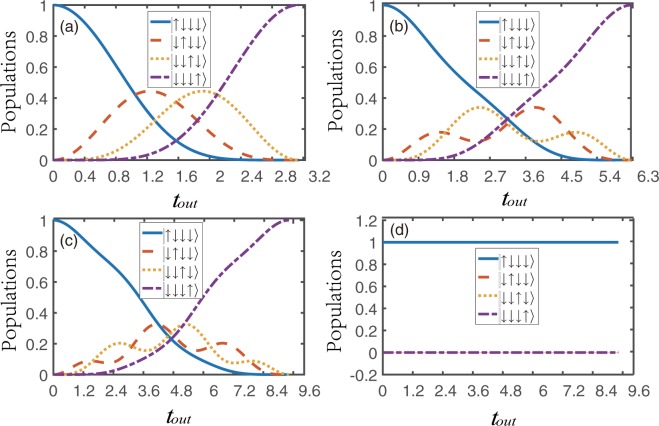
The implementation of quantum spin transistor in the spin chain with *N* = 4 spins. Here and hereafter, Δ, *J*_1_, *h*, *h*_*d*_, *ω*, *a*, *A*_*j*_, *B*_*j*_ and *C*_*j*_ are rescaled in units of *J*_2_, and *t*_*out*_ is then in units of 1/*J*_2_. Hence all parameters are dimensionless. For (**a**–**c**) the parameters are Δ = −1, *J*_2_ = 1, *h* = *h*_0_. The other parameters chosen are *m*_1_ = *m*_2_ = *m*_3_ = 0 for (**a**), *m*_1_ = 1,*m*_2_ = *m*_3_ = 0 for (**b**) and *m*_1_ = 2, *m*_2_ = *m*_3_ = 0 for (**c**). For (**d**) the *h* = *h*_0_ + *h*_*d*_ *sinωt* and other parameters satisfy *h*_*d*_ = 1.2*ω, *m*_1_ = 2, *m*_2_ = *m*_3_ = 0, *J*_2_ = 1, Δ = −1, *ω* = 25.

Moreover, we can get the time interval for transfer, which is ${t}_{out}=\frac{\mathrm{(1}+{m}_{1}+{m}_{3})\pi }{{J}_{2}}$. We find that, there is a relationship between these parameters and the shortest transfer time of the spin excitation between the initial and final states is (*t*_*min*_) = *π*/*J*_2_. When these parameters satisfy the above relation, the system can achieve the perfect state transfer, see Fig. 2(a–c). However, when we add the high frequency periodic driving, it may show that the quantum transmission will be blocked. According to Eq. (8), for sinusoidal driving (*f*_*j*_(*t*) = *A*_*j*_ *sinωt*, *j* = 1,4) the renormalized nearest-neighbour coupling strength takes the form

14 $${J}_{j}^{eff}={J}_{0}(\frac{2({A}_{j+1}-{A}_{j})}{\omega }){J}_{j},{J}_{j}^{eff^{\prime} }=0,$$

where *J*_0_(*x*) is the zeroth order Bessel function of the first kind and *A*_*j*+1_ − *A*_*j*_ is the amplitude difference between adjacent lattice points. The frequency of the time-dependent driving field is described by the parameter *ω*. From expression (14), we immediately observe that the change of renormalized parameters of the system would affect the dynamics of the system. Thus when 2(*A*_*j*+1_ − *A*_*j*_)/*ω* is equal to a zero of *J*_0_ the system’s dynamics is frozen. That is to say, such an initial state will remain stationary and the sinusoidal driving would block the transfer of the spin state between the input and output ports, see Fig. 2(d). However, the results of adding triangular-well driving and square-well driving in the system will be different from the exact ones for 2(*A*_*j*+1_ − *A*_*j*_)/*ω*. Specific details will be analyzed in the following discussions.

Figure 2(a–c) show that the two intermediate states are coupled and a perfect state transfer is achieved between the input and output ports without energy dissipation. When the value of *m*_*j*_ changes, the interaction form and transmission time of the intermediate state change but the perfect state transmission is not affected. From Fig. 2(d), we can learn that the dynamic evolution of the system will be completely frozen when the periodic driving field is added.

**Figure 3:**
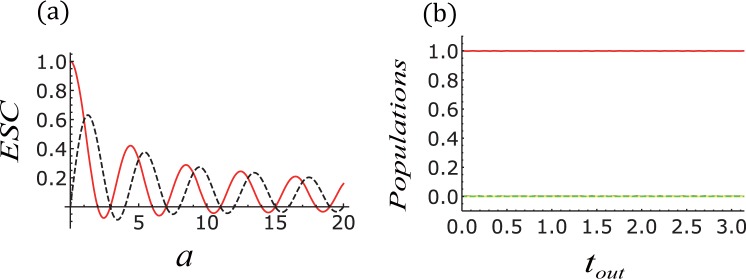
(**a**) The effective coupling strength (*ECS*) vs *a*, given by Eq. (16). (**b**) The system dynamics driven by triangular wave field with *a* = 2.9. And other parameters chosen are *m*_1_ = 2, *m*_2_ = *m*_3_ = 0, *J*_2_ = 1, *ω* = 25, Δ = −1.

Next, with triangle-well drive, the periodic driving field can be written as,

15 $${f}_{j}(t)=\{\begin{array}{ll}\frac{2{B}_{j}\omega }{\pi }t, & 0\le t < \frac{\pi }{2\omega },\\ -\frac{2{B}_{j}}{\pi }(\omega t-\pi ), & \frac{\pi }{2\omega }\le t < \frac{2\pi }{3\omega },\\ \frac{2{B}_{j}}{\pi }(\omega t-2\pi ), & \frac{2\pi }{3\omega }\le t < \frac{\pi }{\omega },\end{array}$$

where j = 1 and 4, *B*_*j*_ and *ω* are the amplitude and frequency of triangle-well driving, respectively. According Eq. (4) we also get the *J*_*eff*_ and ${J}_{eff^{\prime} }$

16 $${J}_{j}^{eff}=\frac{\sqrt{2}f\,\cos (\frac{a\pi }{4})}{\sqrt{a}}{J}_{j},{J}_{j}^{eff^{\prime} }=\frac{\sqrt{2}f\,\sin (\frac{a\pi }{4})}{\sqrt{a}}{J}_{j},$$

where $f=\,\cos (a\pi \mathrm{/4)}FresnelC(\sqrt{a\mathrm{/2}})+FresnelS(\sqrt{a\mathrm{/2}})\sin (a\pi \mathrm{/4)}$, *a* = 2(*B*_*j*+1_ − *B*_*j*_)/*ω*. $FresnelC(x)=$ ${\int }_{0}^{x}cos(\pi {t}^{2}\mathrm{/2)}dt$ and $FresnelS(x)={\int }_{0}^{x}sin(\pi {t}^{2}\mathrm{/2)}dt$ are Fresnel integrals. From Fig. 3, we have two observations: (i) Fig. 3(a) demonstrates the relation between the parameters of *a* and effective coupling strength (*ECS*). When 2(*B*_*j*+1_ − *B*_*j*_)/*ω* ≈ 2.9, 7.0, i.e.,the effective coupling strength is zero, the nearest-neighbour interaction is destroyed. Therefore, the system’s dynamics is frozen see Fig. 3(b). (ii) Of course, when 2(*B*_*j*+1_ − *B*_*j*_)/*ω* tuned far away from those values the effective coupling strength is not zero. They will partially suppress the transmission of the perfect state but does not completely block the transmission.

Finally, the square-well field is another waveform that is availabe experimentally. In fact these driving fields have already been studied in time-periodic driving system. The square-well driving can be expressed as,

17 $${f}_{j}(t)=\{\begin{array}{cc}{C}_{j}, & 0\le t < \frac{T}{2},\\ -{C}_{j}, & \frac{T}{2}\le t < T,\end{array}$$

where j = 1, 4 and *C*_*j*_ is the amplitude of square-well. Similarly, the effective coupling strength of the system is changed to ${J}_{j}^{eff}=\frac{\sin ({C}_{j+1}-{C}_{j})T}{({C}_{j+1}-{C}_{j})T}{J}_{j}$, ${J}_{j}^{eff^{\prime} }=\frac{1-\,\cos ({C}_{j+1}-{C}_{j})T}{({C}_{j+1}-{C}_{j})T}{J}_{j}$. When ${J}_{j}^{eff}={J}_{j}^{eff^{\prime} }=0$, the dynamic transmission system will be completely blocked at this time 2(*C*_*j*+1_ − *C*_*j*_)/*ω* = 2. When the system driver changes, the condition of completely blocked state transmission is also changed. More obviously and straightforwardly, the time evolution of the driven is sensitive to the renormalized quantities.

**Figure 4:**
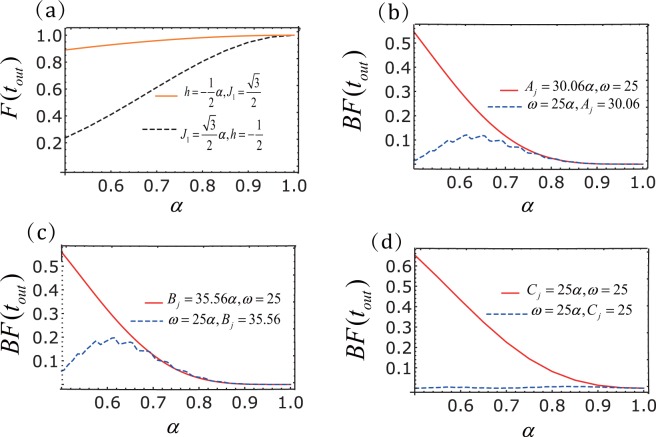
Robustness of the state transfer with *N* = 4. (**a**) The transfer fidelity *F*(*t*_*out*_) of the spin chain. The transfer fidelity *F*(*t*_*out*_) for different values of *h* (orange line) or *J*_1_ (black-dashed line). One of the parameters (*h* or *J*_1_) has a derivation from the ideal value $h=-\,\frac{1}{2}$, ${J}_{1}=\frac{\sqrt{3}}{2}$, while other parameters *J*_2_ = 1, Δ = −1 are keep unchanged. Breaking fidelity of spin chain with sinusoidal driving (**b**), (**c**) triangular-well driving, and (**d**) square-well driving. The blockade fidelity *BF*(*t*_*out*_) for different values of amplitude(red line) and frequency (blue-dashed line), where the ideal parameters are *A*_*j*_ = 30.06, *B*_*j*_ = 36.56, *C*_*j*_ = 25, *ω* = 25.

The perfect transfer(complete blockade) of the target spin for the open(closed) gate as shown in Figs 2 and 3 is obtained for the system parameters satisfying Eq. (12) and Eq. (14). It is important to quantify the sensitivity of the spin transistor to fluctuations of the parameters. In Fig. 4(a) we show the dependence of transfer fidelity on the system parameters.

18 $$F({t}_{out})=|\langle \uparrow \downarrow \downarrow \,\downarrow |{e}^{-i{H}_{eff^{\prime} }{t}_{out}}|\uparrow \downarrow \downarrow \downarrow \rangle {|}^{2}.$$

We observe that coherent transfer is robust against the variations in *h*, but is rather sensitive to small variations in *J*_1_ since it detrimentally affect the resonant conditions. In Fig. 4(b–d) we show the dependence of the blockade fidelity

19 $$BF({t}_{out})=|\langle \uparrow \downarrow \downarrow \,\downarrow |{e}^{-i{H}_{eff}{t}_{out}}|\uparrow \downarrow \downarrow \downarrow \rangle {|}^{2},$$

on the uncertainty of the three driving frequencies and amplitudes. Obviously, the uncertainty of the three driving frequencies has a less effect on the blocking of the gate than amplitudes. The frequency fluctuations of the square-well driving have less influence on the blocking state transmission than the other two drivings.

### The collective dephasing effect in the system

In this part, we mainly discuss the effect of decoherence on the state transfer. We introduce a stochastic Hamiltonian to simulate the decoherence^48^. It reads

20 $$\begin{array}{rcl}{H}_{s}(t) & = & H(t)+{H}_{S0}(t),\\ H(t) & = & \sum _{j=1}^{N}{h}_{j}{\sigma }_{z}^{j}-\frac{1}{2}\sum _{j=1}^{N-1}{J}_{j}[{\sigma }_{x}^{j}{\sigma }_{x}^{j+1}+{\sigma }_{y}^{j}{\sigma }_{y}^{j+1}+{\rm{\Delta }}{\sigma }_{z}^{j}{\sigma }_{z}^{j+1}]+\sum _{j=1}^{N}\,{f}_{j}(t){\sigma }_{z}^{j},\\ {H}_{S0}(t) & = & \sum _{j=1}^{N}\delta \cdot {h}_{j}\eta (t){\sigma }_{z}^{j}-\frac{1}{2}\sum _{j=1}^{N-1}\delta \cdot {J}_{j}\eta (t)[{\sigma }_{x}^{j}{\sigma }_{x}^{j+1}+{\sigma }_{y}^{j}{\sigma }_{y}^{j+1}+{\rm{\Delta }}{\sigma }_{z}^{j}{\sigma }_{z}^{j+1}].\end{array}$$

The Hamiltonian *H*_*s*_(*t*) is composed of the target Hamiltonian *H*(*t*) and the stochastic part *H*_*S*0_. Among them, the stochastic part includes operators ${\sigma }_{x,y,z}^{j}$. A positive real constant *δ* and an stochastic Gauss process *η*(*t*) independent of *δ*. Averaging the random density matrix ${\rho }_{s}(t)=|{{\rm{\Psi }}}_{s}(t)\rangle \langle {{\rm{\Psi }}}_{s}(t)|$ corresponding to *H*_*s*_(*t*) over the fluctuations *δ* and *η*(*t*), we arrive at the following master equation^49^

21 $$\begin{array}{rcl}\frac{d}{dt}\rho (t) & = & -i[H(t),\rho (t)]+{\mathscr{D}}[\rho (t)],\\ {\mathscr{D}}[\rho (t)] & = & -\sum _{j,j^{\prime} =1}^{N}{\delta }^{2}{h}_{j}^{2}[{\sigma }_{z}^{j},[{\sigma }_{z}^{j^{\prime} },\rho (t)]]\\  &  & +\frac{1}{2}\sum _{j,j^{\prime} =1}^{N-1}{\delta }^{2}{J}_{j}^{2}[{\sigma }_{x}^{j}{\sigma }_{x}^{j+1},[{\sigma }_{x}^{j^{\prime} }{\sigma }_{x}^{j^{\prime} +1},\rho (t)]]\\  &  & +\frac{1}{2}\sum _{j,j^{\prime} =1}^{N-1}{\delta }^{2}{J}_{j}^{2}[{\sigma }_{y}^{j}{\sigma }_{y}^{j+1},[{\sigma }_{y}^{j^{\prime} }{\sigma }_{y}^{j^{\prime} +1},\rho (t)]]\\  &  & +\frac{1}{2}{\rm{\Delta }}\sum _{j,j^{\prime} =1}^{N-1}{\delta }^{2}{J}_{j}^{2}[{\sigma }_{z}^{j}{\sigma }_{z}^{j+1},[{\sigma }_{z}^{j^{\prime} }{\sigma }_{z}^{j^{\prime} +1},\rho (t)]]\mathrm{.}\end{array}$$

**Figure 5:**
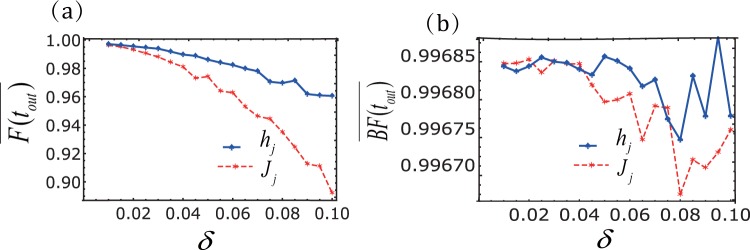
Average fidelity ($\overline{F({t}_{out})}$, $\overline{BF({t}_{out})}$) as a function of noise. The parameters used in this plot are: ${\rm{\Delta }}=-\,1,{J}_{2}=1,{J}_{2}=\sqrt{3}\mathrm{/2},{h}_{1}={h}_{2}=\mathrm{1/2},\omega =25,{A}_{1}=30.06$.

Clearly, this master equation describes the collective decoherence effect of the system. To this extent, we can use the random Hamiltonian in Eq. (20) to simulate the many-body decoherence in the system. As shown in Fig. 5, the effect of noise on the average transmission fidelity $\overline{F({t}_{out})}$ is dramatically, and the effect is much larger than that effect on the average blocking fidelity $\overline{BF({t}_{out})}$. $\overline{F({t}_{out})}$ and $\overline{BF({t}_{out})}$ are defined by $\overline{\langle \uparrow \,\downarrow \,\downarrow \,\downarrow \,|\rho ({t}_{out})|\,\downarrow \,\downarrow \,\downarrow \,\uparrow \,\rangle \,}$ and $\overline{\langle \downarrow \,\downarrow \,\downarrow \uparrow \,|\rho ({t}_{out})|\,\uparrow \,\downarrow \downarrow \downarrow \,\rangle \,}$, respectively.

### Spin Transistor with Longer Chains

Our analysis above is given for *N* = 4 systems as an example to study the behaviors of the quantum transfer in single excitation subspace. Similar behavior can be obtained for longer chains. The Hamiltonian Eq. (3) in this single excitation subspace can be written in a tridiagonal form. And the *N*-particle coupling matrix elements are given by:

22 $$\begin{array}{rcl}\langle m|\tilde{H}(t)|m\rangle  & = & {h}_{m}-\sum _{j\ne m}^{N}{h}_{j}+\frac{{\rm{\Delta }}}{2}({J}_{m-1}+{J}_{m}-\sum _{j\ne m,m-1}^{N-1}{J}_{j}),\\ \langle m-\mathrm{1|}\tilde{H}(t)|m\rangle  & = & -{J}_{m-1}{e}^{-2i({F}_{m}(t)-{F}_{m-1}(t))},\\ \langle m+\mathrm{1|}\tilde{H}(t)|m\rangle  & = & -{J}_{m}{e}^{2i({F}_{m+1}(t)-{F}_{m}(t))},\end{array}$$

where $|m\rangle$, $m=1,2,3,\mathrm{...},N$ refers to a state with a single excitation at site *m*, F_*m*_(*t*) represents the periodic driving of the *m* site. And *h*_*m*_ stands for the magnetic field of the *m* site and *h*_*j*_ represents the magnetic field of the other lattice points outside the *m* site. *J*_*j*_ represents the coupling coefficient except for *J*_*m*−1_ (the coupling coefficient between the *m* − 1 lattice point and the *m* lattice point) or *J*_*m*_ (the coupling coefficient between the *m* lattice point and the *m* + 1 lattice point). By reflecting symmetry, we mean for *F*_*m*_(*t*) = 0, *J*_*j*_ = *J*_*N*−j_ and *h*_*j*_ = *h*_*N*+1−*j*_ wit*h h*_*j*_ = *h*_*N*_ = 0. Thus, the Hamiltonian has double symmetries, the main diagonal and the second diagonal are symmetric. The condition in Eq. (7) is not only sufficient but also necessary for prefect state transfer in mirror-symmetric chains. And the task of solving Eq. (7) is an inverse eigenvalue problem^50,51^, which is usually treated numerically. In^50^, they show how to realize the perfect quantum state transfer and construct a valid two-qubit gates between bosonic and fermionic networks. And in^51^, they achieve perfect transmission and the problem of speed limit for QST is put forward at the same time. However, we can still achieve perfect state transmission by using different Hamiltonian. And when the system contains periodic driving, it can achieve a complete blockade of transmission. In our work, we find the simplest solution to Eq. (7)

23 $$\begin{array}{rcl}{h}_{m} & = & \frac{1}{2}(C-{\rm{\Delta }}({J}_{m-1}+{J}_{m})+E+\frac{1}{2}{\rm{\Delta }}F),\\ {J}_{m} & = & \sqrt{m(N-m)},\end{array}$$

where *C* is a constant, $E={\sum }_{j=1}^{N}{h}_{j}$, $F={\sum }_{j=1}^{N-1}{J}_{j}$ and $E=\frac{(\frac{{\rm{\Delta }}}{2}N-2{\rm{\Delta }})}{2-N}F$. Similarly, when *F*_*m*_(*t*) ≠ 0 we can effectively block the transmission of the particles.

**Figure 6:**
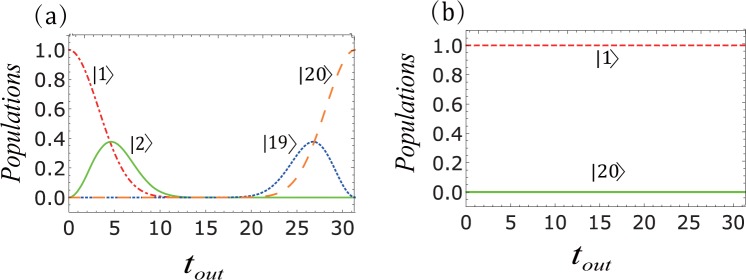
Transmission and blockage in the spin chain with *N* = 20. (**a**) The populations in twenty-site system and the corresponding parameters satisfies: *t*_*out*_ = *Nπ*/2, ${J}_{m}=\sqrt{m(N-m)}$, where j = 1, 2, 19, 20. Among them, the red dot-dashed and orange dashed lines represent the populations of the initial and final state; Similarly, in (**b**) the red dashed line and the green solid line represents the probability of particles in the initial state and final state. The parameters chosen are *t*_*out*_ = *Nπ*/2, *ω* = 25 and *A*_1_ = 30.06.

In Fig. 6(a), we show the perfect state transfer of *N* = 20. Furthermore, the process of using sinusoidal driving to block quantum state transfer is shown in Fig. 6(b). We observe that the triangle-well driving and the square-well driving are similar to the sinusoidal driving.

**Figure 7:**
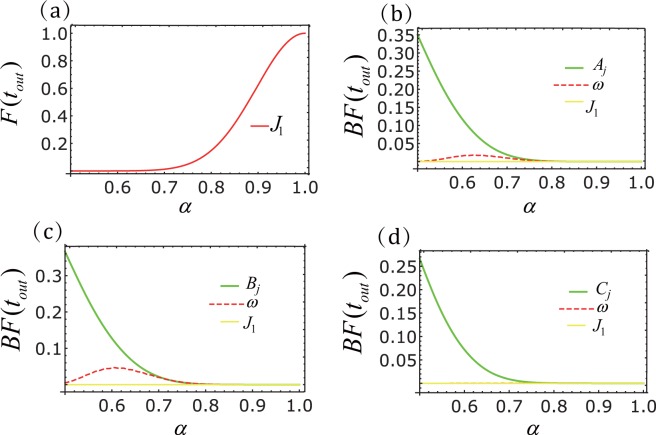
Performance versus fluctuations (*N* = 20). (**a**) The transmission fidelity *F*(*t*_*out*_) of spin chain. The transfer fidelity *F*(*t*_*out*_) of ${J}_{1}=\sqrt{19}\alpha$. (**b**–**d**) Blockade fidelity of spin chain with sinusoidal driving, triangular-well driving and square-well driving, respectively. The blockade fidelity *BF*(*t*_*out*_) for different values of amplitude(green line), frequency (red-dashed line) and *J*_1_, where *A*_*j*_ = 30.06*α*, *B*_*j*_ = 36.56*α*,*C*_*j*_ = 25*α*, *ω* = 25*α*, ${J}_{1}=\sqrt{19}\alpha$ and the ideal parameters are *A*_*j*_ = 30.06, *B*_*j*_ = 36.56, *C*_*j*_ = 25, *ω* = 25, ${J}_{1}=\sqrt{19}$. Here *A*_*j*_, *B*_*j*_, *C*_*j*_, *ω*, *J*_1_ are amplitude, frequency and interaction coefficient between adjacent particles, respectively.

Next, we will discuss the fidelity of the longer chain. In Fig. 7 we show the effect of some parameters on the fidelities of *N* = 20. We observe that the perfect state transmission is sensitive to variations in *J*_1_. This is obvious because the resonance conditions in the longer spin transistors are affected. Conversely, because the state transfer blockade depends on the larger energy mismatch, it is not affected by the uncertainty of the *J*_1_. At the same time, from Fig. 7(b) to (d) we can also see that the impact of amplitude uncertainty is still greater than that of frequency uncertainty. More importantly, as the number of particles increases, the uncertainty of the system parameters decreases the effect of the state blocking.

## Discussion

In conclusion, a scheme for state transfer and blockage has been proposed in one dimension Heisenberg spin chain. In our scheme, the absence or presence of a periodic driving potential can allow or block the transfer of an arbitrary spin state between the input and output ports. In the absence of periodic driving, we obtain the analytic solutions for the system, leading to the condition of the perfect state transfer. In the case of periodic driving, we deduce the effective Hamiltonian and analyze separately three different drives to achieve complete blockage. We also analyze the sensitivity of spin transistor (*F*(*t*_*out*_) and *BF*(*t*_*out*_)) to the fluctuation in parameters and examine the effect of decoherence on the state transfer. Our system realizes a control of quantum state transfer that can be more convenient and concise. In other words, we can control the transmission of the quantum state at any time with high fidelity, which can be treated as a spin transistor. This might be of great interests to integrated quantum logic elements and quantum information processing.
